# Influence of a family history of type 2 diabetes, demographic and clinical data on carotid intima-media thickness in patients with type 1 diabetes: a cross-sectional study

**DOI:** 10.1186/1475-2840-13-87

**Published:** 2014-05-03

**Authors:** Carlos Roberto Moraes de Andrade, Eliete Leão Clemente Silva, Maria de Fátima Bevilaqua da Matta, Marcia Bueno Castier, Maria Luiza Garcia Rosa, Marília de Brito Gomes

**Affiliations:** 1 Endocrinology Department at University Hospital of Fluminense Federal University, Rio de Janeiro, Brazil, and at Diabetes Unit, State University Hospital of Rio de Janeiro, Rio de Janeiro, Brazil; 2 Technical Nursing at Department of Internal Medicine, Diabetes Unit, State University Hospital of Rio de Janeiro, Rio de Janeiro, Brazil; 3 Biologist at Department of Internal Medicine, Diabetes Unit, State University Hospital of Rio de Janeiro, Rio de Janeiro, Brazil; 4 Department of Internal Medicine, Cardiology Unit, State University Hospital of Rio de Janeiro, Rio de Janeiro, Brazil; 5 Epidemiology and Biomaths at University Hospital of Fluminense Federal University, Rio de Janeiro, Brazil; 6 Department of Internal Medicine, Diabetes Unit, State University Hospital of Rio de Janeiro, Rio de Janeiro, Brazil; 7 Rua Ignez Peixoto 565 casa 6, Itaipú, Niterói, Rio de Janeiro, Brazil

**Keywords:** Type 1 diabetes, Carotid IMT, CVD risk, Women with type 1 diabetes, Family history of type 2 diabetes

## Abstract

**Background:**

Intima-media thickness (IMT) of the common carotid artery is a surrogate end point of cardiovascular disease (CVD). Identifying the factors associated with a higher IMT may contribute to the identification of subjects with higher CVD risk. Our objective was to compare the common carotid IMT of type 1 diabetes patients to healthy control subjects. The secondary objective was to determine factors associated with a higher carotid IMT.

**Methods:**

We conducted a cross-sectional study between March 2009 and October 2013, comprising 127 type 1 diabetes patients and 125 control subjects matched by age, gender and body mass index (BMI). Carotid IMT was measured using semi-automated edge detection software.

**Results:**

Type 1 diabetes patients had a higher median IMT compared with control subjects (0.538; IQR: 0.500-0.607 *vs* 0.513 mm; IQR: 0.481-0.557, respectively p = 0.001). Women with type 1 diabetes had a higher median IMT difference compared to the control group (0.537; IQR: 0.495-0.596 *vs* 0.502 mm; IQR: 0.472-0.543, respectively p = 0.003) than did men with type 1 diabetes (0.547; IQR: 0.504-0.613 *vs* 0.528 mm; IQR: 0.492-0.575, respectively p = 0.2). Age and diabetes duration had an additive effect on the IMT of type 1 diabetes patients. Multivariate gamma regression model analysis showed that in type 1 diabetes patients, the IMT was associated with age (Exp (β) = 1.006, p < 0.001), duration of diabetes (Exp (β) = 1.004, p = 0.001), BMI (Exp (β) = 1.005, p = 0.021), family history of type 2 diabetes (Exp (β) = 1.044, p = 0.033), total cholesterol (Exp (β) = 0.999, p = 0.001) and creatinine clearance (Exp (β) = 1.000, p = 0.043).

**Conclusions:**

Patients with type 1 diabetes have increased IMT, a marker of subclinical atherosclerosis. The CVD risk may be similar between men and women with type 1 diabetes, suggesting a loss of gender protection. Also, CVD risk may be higher in those with a family history of type 2 diabetes. Prospective studies are needed to confirm the predictive value of these findings and the causal effect between IMT and CVD in patients with type 1 diabetes.

## Background

There is a worldwide increase in type 1 diabetes incidence [1,2]. The disease can evolve to chronic complications, resulting in higher morbidity and mortality and consequently higher costs [1-3]. Furthermore, there has been an increase in the prevalence of overweight and obese patients in the type 1 diabetes population all over the world, including Brazil [4,5].

The cardiovascular disease (CVD) risk in type 1 diabetes is approximately 10 times higher than that in the general population, even in the absence of classical risk factors and adequate metabolic control [6-15]. Type 1 diabetes patients are continuously exposed to a hyperglycemic environment and other CVD risk factors, such as insulin dosage and the presence of chronic complications [16]. All of these risk factors are present at a very young age, resulting in progressive endothelial dysfunction that results in the atherosclerosis process [12,14,15]. One study showed that the soluble form of the membrane glycoprotein CD146 (sCD146) derived from endothelium cells is increased in individuals with cardiovascular and inflammatory disease and is associated with endothelial dysfunction [17]. Even in type 1 diabetes patients without chronic complications there is a mild chronic inflammatory state, which can be observed as an elevation of acute phase proteins (such as C-reactive protein (CRP)) that may also contribute to atherosclerosis [18]. Furthermore, some other unknown CVD risk factors might be present as there is still a residual CVD risk even in patients with excellent metabolic control [16].

More accurate methods for detection of sub-clinical atherosclerosis would be useful for identifying patients with type 1 diabetes with high CVD risk. The complex involving the intima and media layer thickness (IMT) of the common carotid artery measured by ultrasound is related to subclinical atherosclerosis process and higher CVD risk in type 1 diabetes patients [7,19-21]. Furthermore, it has been suggested that there is a linear correlation between the increase in total and cardiovascular death and the increase in IMT [22-25]. This measurement is a simple and non-invasive procedure [22,26,27].

Intensive glucose control is able to postpone the IMT increase, although it does not prevent it [6,12]. The DCCT/EDIC study showed that though both the original intensive and conventional treatment groups had the same glycated hemoglobin (HbA1c) during the 12 years of the EDIC study, the original intensive treatment group still had a thinner IMT [28]. Many factors may influence the IMT and, in fact, there may be some unrecognized factors associated with it [12,13,24,29-33].

The primary objective of this study was to compare the carotid IMT between type 1 diabetes patients and healthy control. The secondary objective was to determine the clinical and laboratorial variables that are associated with a thicker carotid IMT in these populations.

## Methods

This was a cross-sectional, single center study conducted between March 2009 and October 2013 in consecutive patients with type 1 diabetes who regularly attended a tertiary care diabetes outpatient unit at Pedro Ernesto University Hospital. These patients were matched to healthy control subjects by age, gender and body mass index (BMI). Age was matched by age group with a range of five years because of our difficulty in finding older healthy control subjects. Control subjects were recruited among the patients’ spouses and relatives, university students and hospital employees. The study was approved by the local research ethics committee of Pedro Ernesto University Hospital, Rio de Janeiro State Univerity, Rio de Janeiro, Brazil. Participants were subjected to clinical and laboratory evaluation.

Initially, written informed consent was obtained from all participants. Subjects were submitted to an interview for demographic and clinical information and underwent physical examination. On the morning of the interview, subjects brought in the first 10- hour overnight urine sample, and fasting and post-prandial blood samples were collected. Subjects were then conducted to the Echo laboratory acclimatized room to perform the IMT measurement.

The inclusion criteria included individuals older than 10 years, patients with type 1 diabetes for more than five years, patients who continuously used insulin since diagnosis and healthy control subjects. The exclusion criteria included type 1 diabetes patients and healthy control subjects who were unable to tolerate dorsal decumbency for long periods, a previous history of invasive procedure in the carotid artery, chronic usage of glucocorticoids, asthma or chronic obstructive pulmonary disease, kidney disease, liver failure, thyroid disease and control subjects with previous histories of high cholesterol levels or using cholesterol-lowering drugs, hypertension or using pressure-lowering drugs and cardiovascular disease.

We followed the American Diabetes Association (ADA) statement for the definition of childhood and adolescence. [34]. BMI was calculated by dividing the weight in kilograms by the squared height in meters. Overweigh was defined as a BMI ≥ 25 for adults or ≥ 85^th^ percentile for children, and obesity was defined as a BMI ≥ 30 for adults or ≥ 95^th^ percentile for children and adolescents [35].

Blood pressure (BP) was calculated using the mean of the three measurements. Adult subjects were classified as having hypertension when the mean was higher than 140 mmHg for systolic blood pressure (SBP) and/or 90 mmHg for diastolic blood pressure (DBP) [36,37]. Children and adolescents were considered to have hypertension if SBP or DBP was ≥ 95^th^ percentile for age, sex and height [35].

Glucose control was assessed by fasting glucose (FG) and HbA1c (high performance liquid chromatography (HPLC), Bio-Rad Kit, hemoglobin testing system equipment from Bio-Rad Lab., Irvine, USA. Reference values = 4.0 to 6.0%).

Uric acid, serum creatinine, triglycerides, High density cholesterol (HDL) and total cholesterol levels were measured by enzymatic techniques (Cobas Mira; Roche, Bohemia, NY, USA). Low density cholesterol (LDL) was calculated using the Friedewald equation, except when the triglyceride levels were higher than 400 mg/dL [38]. Creatinine clearance was calculated using the Cockcroft-Gault equation. Urinary albumin excretion rate (UAER) was estimated by solid-phase competitive chemiluminescent enzyme immunoassay (sensitivity of 0.5 mcg ⁄mL; Immulite 1000 Systems; DPC Medlab, Los Angeles, CA, USA) with intra- and inter-assay variation coefficients of 4.4 and 6.1%, respectively. Serum CRP was measured using a highly sensitive immunonephelometry assay (Behring Nephelometer; Behring, Marburg, Germany) with a detection limit of 0.01 mg/dl and intra- and inter-assay variation coefficients of 1 and 5.3%, respectively.

The IMT image was digitally recorded using a commercially available system (Envisor CHD, Philips, Bothell, WA, USA) equipped with a linear L12-13Hz transducer. The IMT was measured using the semi-automated edge-detection software package Q-LAB Advanced Ultrasound Quantification Software version 7.1, Philips. This software measured the IMT in millimeters with a three decimal places precision. Measurements were obtained according the American Society of Echocardiography recommendations [26].

A single examiner performed all IMT images in a quiet, dark, acclimatized room, after the patient rested for at least five minutes. A bilateral transversal scanning from the common carotid artery and its visible ramifications was performed to look for apparent plaques. The distal one centimeter length of the common carotid IMT image was stored in a digital media after positioning the transducer longitudinal to the carotid vessel. The images were taken in three different angles, posterior, lateral and anterior, for each right and left common carotid artery. The IMT was measured off-line using the semi-automated edge-detection software by two independent skilled examiners who were blinded for the condition of the subject analyzed.

Statistical analysis was performed using the SPSS software version 21 (IBM Corp., USA). To detect a 0.021 mm difference in the IMT between type 1 diabetes and control subjects with a statistical power of 80%, we would need 120 individuals in each group. To determine the agreement between both examiners, we calculated the intra-class coefficient (95% CI) and performed the Bland-Altman box-plot. Data are presented in median and interquartile range (IQR). The non-parametric data were analyzed using the Mann–Whitney *U* test or the unpaired t test when applicable. The bivariate analysis was performed using gamma regression model between the IMT and the variables analyzed. Those variables with a *p* value < 0.1 in the bivariate model were included in the multivariate gamma regression model, which was performed in backwards. To avoid multicollinearity, when two or more variables were a measure of the same risk factor, we chose the most significant one. We performed the multivariate gamma model analysis in backwards because there were a lot of variables to be analyzed.

## Results

### Overview of demographic and laboratorial data of the studied population

Characteristics of the study population are shown in Table 1. In patients with type 1 diabetes, age ranged from 14 to 63 years old and in the control group, age ranged from 14 to 57 years old. There was only one patient with type 1 diabetes who was older than 57 years. Compared to the control group, patients with type 1 diabetes had lower alcohol consumption (9.6 *vs* 10.4%, p < 0.001), higher median FG (117 mg/dL; IQR: 78–260 *vs* 78 mg/dL; IQR: 70.5-85.5, p < 0.001), higher median HbA1c (8.9%; IQR: 7.8-10.5 *vs* 5.4%; IQR: 5.2-5.8, p < 0.001), lower median creatinine (0.86 mg/dL; IQR: 0.7-1.0 *vs* 0.93 mg/dL; IQR: 0.77-1.09, p = 0.007), lower median triglycerides (74.5 mg/dL; IQR: 57–108.8 *vs* 91 mg/dL; IQR: 68–129.5, p = 0.05), lower median uric acid (3.7 mg/dL; IQR: 2.9-4.5 *vs* 5.1 mg/dL; IQR: 4.0-6.3, p < 0.001) and higher median UAER (8.56 mcg/min; IQR: 4.31-25.33 *vs* 3.39 mcg/min; IQR: 2.11-5.99, p < 0.001).

**Table 1:** Clinical and laboratory data in patients with type 1 diabetes and control subjects

	**Type 1 diabetes**	**Control**	**P**
**Demographic data**			
N (% male)	127 (45.7)	125 (40.8)	0.4
Age (years)	32 (23–42)	29 (22–39.5)	0.08
Age at diagnosis (years)	15 (10–22)		
Duration of diabetes (years)	14 (10–21.8)		
Past history of hypertension (%)	24.8%		
Current smoke (%)	9.6	10.4	0.8
Current alcohol consumption (%)	30.4	58.3	<0.001
Current exercise practice (%)	42,4	50.6	0.3
**Family history**			
Family history of type 2 diabetes (%)	29,8	25.2	0.5
Family history of type 1 diabetes (%)	19.2	18.3	0.9
Family history of hypertension (%)	64	52.2	0.07
Family history of CVD (%)	21	13	0.1
**Anthropometric data and insulin dose**			
BMI (kg/m^2^)	23.6 (21.6-26.2)	24.3 (21.6-28)	0.2
SBP (mmHg)	120 (110–125.8)	113 (105–120)	0.06
DBP (mmHg)	70 (70–80)	70 (68.5-80)	0.2
Waist (cm)	81 (74–88)	81 (73.1-89.9)	0.9
WHR	0.86 (0.8-0.9)	0.85 (0.79-0.91)	0.5
Insulin/weight (U/Kg)	0.81 (0.59-1.07)		
**Laboratory data**			
Fasting glucose (mg/dL)	117 (78–260)	78 (70.5-85.5)	<0.001
HbA1c (%)	8.9 (7.8-10.5)	5.4 (5.2-5.8)	<0.001
Creatinine (mg/dL)	0.86 (0.7-1.0)	0.93 (0.77-1.09)	0.007
Creatinine clearance (mL/min)	112.7 (88.5-131.3)	103.5 (87.4-119.4)	0.1
Uric acid (mg/dL)	3.7 (2.9-4.5)	5.1 (4.0-6.3)	<0.001
Total cholesterol (mg/dL)	163 (148–189)	167.5 (146–197.8)	0.3
Triglycerides (mg/dL)	74.5 (57–108.8)	91 (68–129.5)	0.05
HDL (mg/dL)	55.7 (44.7-67.4)	54.9 (44.1-65.8)	0.3
LDL (mg/dL)	90.1 (74.9-105.3)	96 (73.3-115.5)	0.07
CRP (mg/dL)	0.144 (0.066-0.340)	0.120 (0.030-0.320)	0.2
UAER (mcg/min)	8.56 (4.31-25.33)	3.39 (2.11-5.99)	<0.001
**IMT data**			
IMT on the right (mm)	0.533 (0.497-0.600)	0.513 (0.478-0.555)	0.002
IMT on the left (mm)	0.530 (0.490-0.610)	0.510 (0.475-0.562)	0.005
Total IMT (mm)	0.538 (0.500-0.607)	0.513 (0.481-0.557)	0.001

*N* = Number, *CVD* = Cardiovascular disease, *BMI* = Body mass index, *SBP* = Systolic blood pressure, *DBP* = Diastolic blood pressure, *WHR* = Waist to hip ratio, U = International units, HbA1c = Glycated hemoglobin, *HDL* = High density lipoprotein, *LDL* = Low density lipoprotein, *CRP* = C reactive protein, *UAER* = Urinary Albumin excretion rate, *IMT* = intima media thickness. Data are presented in median and interquartile range (IQR).

Among patients with type 1 diabetes, 74.8% had diabetes duration ≥ 10 years, and 32.3% had a diabetes duration ≥ 20 years. There were 45.9% with HbA1c > 9% and 11.9% with HbA1c ≤ 7%. Additionally, in patients with the type 1 diabetes, there were 15% of subjects with a BP ≥ 140×90 mmHg or over the 95% percentile for children and adolescents, 9.6% with a current history of smoking, 33.1% with BMI of ≥ 25 kg/m^2^ or over the 85% percentile for children and adolescents, 7.9% with a HDL < 40 mg/dL for men or < 50 mg/dL for women, 27.9% with a LDL > 100 mg/dL, 33.9% with a CRP >0.3 and 21.3% with an UAER > 30 mcg/min.

Among the control group, 10.4% subjects had current history of smoking, 47% with a BMI of ≥ 25 or over the 85% percentile for children and adolescents, 12% with a HDL < 40 mg/dL for men or < 50 mg/dL for women, 41% with a LDL > 100 mg/dL and 33.3% with CRP > 0.3. There were no control subjects with an UAER ≥30 mcg/min or BP ≥ 140×90 mmHg or over the 95% percentile for children and adolescents.

### Overview of IMT agreement between the two examiners and difference between right and left IMT

**Figure 1:**
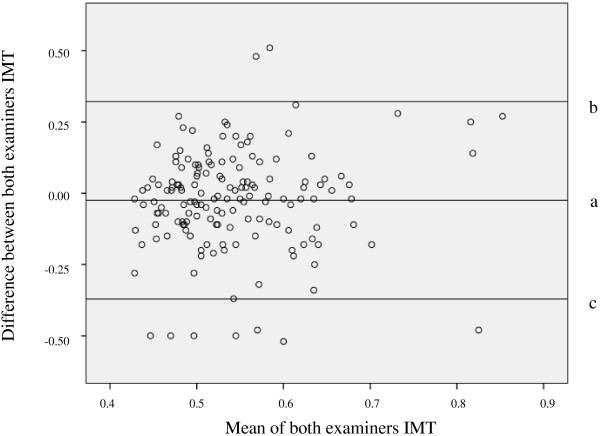
**Bland**-**Altman agreement boxplot between examiners.** Lines: a **=** mean difference of IMT, b = +1.96SD mean difference of IMT, c = -1.96SD mean difference of IMT. IMT = intima media thickness.

There was a high agreement between both independent analyzers using the intra-class coefficient (0.963 95% CI = 0.950-0.973, p < 0.001) (Figure 1).

There was no difference between the right and left mean IMT in any group, as shown in Table 2. Therefore the total mean IMT was employed for statistical analysis.

**Table 2:** Comparison between right and left IMT measurements in patients with type 1 diabetes and control subjects

	**Right IMT (mm)**	**Left IMT (mm)**	**Difference (mm)**	**p**
Type 1 diabetes	0.533 (0.497-0.600)	0,530 (0.490-0.610)	0.003	0.7
Control	0.513 (0.478-0.555)	0.510 (0.475-0.562)	0.003	0.9

*IMT* = Intima-Media Thickness. Data are presented in median and interquartile range (IQR).

### Overview of IMT data of the whole population

Type 1 diabetes patients had higher median right, left and total carotid IMT compared with the control subjects (0.533 mm; IQR: 0.497-0.600 *vs* 0.513 mm; IQR: 0.478-0.555, p = 0.002, 0.530 mm; IQR: 0.490-0.610 *vs* 0.510 mm; IQR: 0.475-0.562, p < 0.005 and 0.538 mm; IQR: 0.500-0.607 *vs* 0.513 mm; IQR: 0.481-0.557, p = 0.001, respectively). The data are shown in Table 1.

**Figure 2:**
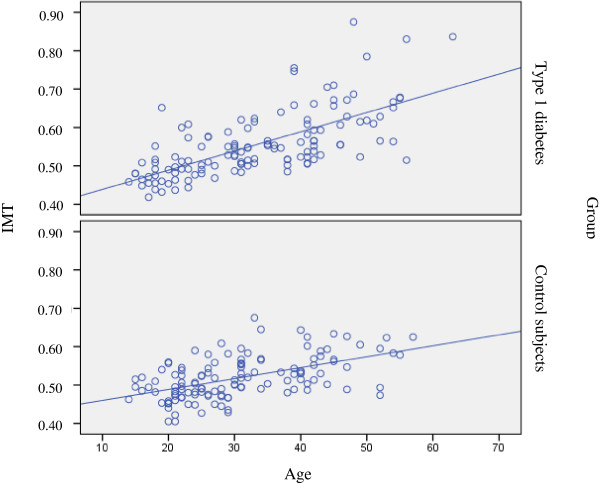
**Association of IMT with age in control subjects and patients with type 1 diabetes.** IMT = intima media thickness.

The difference in median IMT between patients with type 1 diabetes and the control group was higher in women (0.537 mm; IQR: 0.495-0.596 *vs* 0.502 mm; IQR: 0.472-0.543, respectively p = 0.003) than in men (0.547 mm; IQR: 0.504-0.613 *vs* 0.528 mm; IQR: 0.492-0.575, respectively p = 0.2). The influence of age on carotid IMT in type 1 diabetes patients and control subjects is shown in Figure 2. The variables associated with IMT in the bivariate gamma regression model are listed in Table 3.

**Table 3:** Bivariate gamma regression model between IMT and clinical and laboratory data

	**Type 1 Diabetes**		**Control**	
	**Exp (β) (95% CI)**	**p**	**Exp (β) (95% CI)**	**p**
**Demographic data**				
Age	1.009 (1.007-1.011)	<0.001	1.005 (1.004-1.007)	<0.001
Gender (men *vs* women)	1.036 (0.981-1.093)	0.202	1.048 (1.010-1.087)	0.013
Duration of diabetes	1.010 (1.008-1.012)	<0.001		
Diagnosis of hypertension	1.133 (1.076-1.193)	<0.001		
Current smoke	1.040 (0.998-1.096)	0.134	1.029 (0.969-1.093)	0.347
Current alcohol consumption	0.975 (0.925-1.027)	0.335	1.001 (0.963-1.040)	0.979
Current exercise practice	0.991 (0.937-1.048)	0.757	0.993 (0.956-1.032)	0.731
**Family history**				
Family history of type 2 diabetes	1.058 (1.002-1.118)	0.042	1.073 (1.027-1.120)	0.001
Family history of type 1 diabetes	1.038 (0.271 to 1.109)	0.273	1.010 (0.961-1.063)	0.685
Family history of hypertension	1.112 (1.054-1.173)	<0.001	1.020 (0.981-1.060)	0.322
Family history of CVD	1.108 (1.036-1.185)	0.003	1.021 (0.967-1.077)	0.458
**Anthropometric data**				
SBP	1.004 (1.002-1.006)	<0.001	1.002 (1.001-1.004)	0.008
DBP	1.003 (1.001-1.005)	0.001	1.003 (1.001-1.005)	0.008
BMI	1.012 (1.005-1.018)	<0.001	1.007 (1.003-1.012)	0.001
Waist	1.004 (1.002-1.007)	0.001	1.004 (1.002-1.005)	<0.001
Hip	1.002 (0.999 to 1.005)	0.161	1.003 (1.001-1.005)	<0.001
WHR	1.811 (1.220-2.691)	0.003	1.465 (1.121-1.915)	0.005
**Laboratory data**				
Fasting glucoses	1.000 (1.000-1.000)	0.622	1.001 (1.000-1.002)	0.185
Glycated hemoglobin	0.987 (0.974-0.999)	0.041	1.054 (1.002-1.110)	0.043
Creatinine	1.026 (1.017-1.034)	<0.001	1.045 (0.960-1.136)	0.311
Creatinine clearance (mg/min)	0.999 (0.998-1.000)	0.001	1.000 (1.000-1.001)	0.410
Total cholesterol	0.999 (0.998-1.000)	0.050	1.001 (1.000-1.001)	0.005
Triglycerides	1.000 (1.000-1.000)	0.809	1.000 (1.000-1.001)	0.006
LDL	1.000 (0.999- 1.001)	0.626	1.001 (1.000-1.001)	0.009
HDL	0.998 (0.997-1.000)	0.018	1.000(0.999-1.001)	0.682
Uric acid	0.993 (0.974-1.012)	0.460	1.008 (0.995-1.021)	0.219
CRP	0.974 (0.879-1.078)	0.607	1.024 (0.975-1.075)	0.343
UAER	1.000 (1.000-1.000)	0.135	1.000 (0.998-1.002)	0.931

Exp (β) = Exponential beta coefficient; *CI* = Confidence interval; *CVD* = Cardiovascular disease; *SBP* = Systolic blood pressure; *DBP* = Diastolic blood pressure; *BMI* = Body mass index; *WHR* = Waist to hip ratio; *LDL* = Low density lipoprotein; *HDL* = High density lipoprotein; *CRP* = *C* reactive protein; *UAER* = Urinary albumin excretion rate.

Multivariate gamma regression analysis in patients with type 1 diabetes showed that having a family history of type 2 diabetes increased the IMT by 4.4%. For each year of age, there was a 0.6% increase in IMT, for each year of diabetes duration, there was a 0.4% increase in IMT and for each one point increase in BMI there was a 0.5% increase in IMT. For each increase of 1 mg/dL in total cholesterol, there was a 0.1% reduction in IMT, and for each 1 mg/min increase in creatinine clearance, there was an almost 0.0%, but statistically significant increase in IMT, as shown in Table 4 and Figure 2.

**Table 4:** Multiple gamma regression model between IMT and clinical and laboratory data

	**Type 1 Diabetes**		**Control**	
	**Exp (β) (95% CI)**	**p**	**Exp (β) (95% CI)**	**p**
Age	1.006 (1.004-1.008)	<0.001	1.004 (1.003-1.006)	<0.001
Gender (Male *vs* Female)	1.029 (0.993-1.066)	0.1	1.054 (1.020-1.088)	0.001
Duration of diabetes	1.004 (1.002-1.007)	0.001		
BMI	1.005 (1.001-1.010)	0.021	1.004 (1.000-1.008)	0.035
Family history of type 2 diabetes	1.044 (1.003-1.085)	0.033	0.049 (1.012-1.088)	0.009
Creatinine clearance	1.000 (0.999-1.000)	0.0043		
Total cholesterol	0.999 (0.999-1.000)	0.001		

Exp (β) = Exponential beta coefficient, 95% *CI* = 95% Confidence interval, *BMI* = Body mass index.

**Figure 3:**
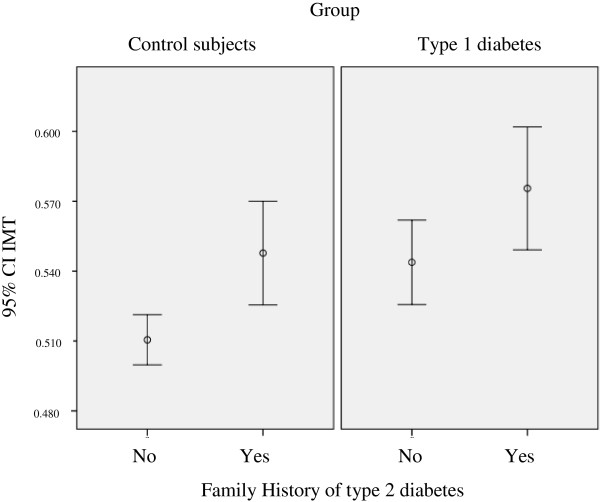
**IMT and family history of type 2 diabetes in control subjects and patients with type 1 diabetes.** CI = confidence interval, IMT = intima media thickness.

Multivariate gamma regression model analysis in the control group IMT showed that the male gender was associated with a 5.4% increase in IMT, having a family history of type 2 diabetes was associated with a 4.9% increase in IMT. For each year of age, there was a 0.4% increase in IMT and for each 1 point increase in BMI, there was a 0.4% increase in IMT. Data are shown in Table 4 and Figures 2 and 3.

Comparing patients with type 1 diabetes with or without family history of type 2 diabetes there was no difference in median BMI (24.1 kg/m^2^; IQR: 22.5-27.45 *vs* 23.5 kg/m^2^; IQR: 21–25.3, respectively, p = 0.08) or in median LDL (93.5 mg/dL; IQR: 80.8-113.4 *vs* 84.6 mg/dL; IQR: 71.9-98, respectively, p = 0.07). Comparing control subjects with or without family history of type 2 diabetes there was no difference in BMI (25.6 kg/m^2^; IQR: 22.7-28.1 *vs* 24.3 kg/m^2^; IQR: 21.5-28.2, respectively, p = 0.5) or in median LDL (100.9 mg/dL; IQR: 88.3-129.8 *vs* 94 mg/dL; IQR: 69.7-108.8, respectively, p = 0.1).

## Discussion

Our study showed that in our sample, patients with type 1 diabetes had a higher IMT compared with the control subjects. Despite having type 1 diabetes for a relatively long time, our patients with type 1 diabetes had a lower IMT than expected, similar to the findings of another study [13]. In our study, in patients with type 1 diabetes, having a family history of type 2 diabetes was associated with a 4.4% increase in carotid IMT and unlike the control group, women with type 1 diabetes had a IMT as high as men.

In the control group, the female gender was associated with a thinner IMT, but this association was not found in our patients with type 1 diabetes. A study that evaluated the distribution of IMT according to age in the general population showed that men had a higher IMT compared with women [39]. These findings could reflect a loss of gender protection for CVD risk in women with type 1 diabetes as suggested by The Diabetes UK Cohort Study and another study [40,41]. In fact, in patients with type 1 diabetes, women may have a higher increase in CVD risk compared with men [8]. In a cohort of 23,000 patients with type 1 diabetes the CVD risk in women with diabetes was not only greater than that for women without diabetes but was also considerably higher than that for men without diabetes [40]. Another study demonstrated that women had a higher arterial stiffness measured at the carotid and radial arteries compared to men [42]. The EURODIAB PCS found that in patients with type 1 diabetes, the CVD risk may be increased approximately 9–29 times in women and 4–9 times in men compared to individuals without diabetes [43].

The higher IMT found in our patients with type 1 diabetes was not associated with current parameters of glycemic control. This findings are similar to the findings in the DCCT/EDIC study, in which carotid IMT was only associated with the HbA1c six years before the IMT measurement [28]. This delayed effect suggests a glycemic memory that favors an early and intensive metabolic control [44].

Among all of the factors associated with IMT, age may be a major one and in our study it was associated with a higher IMT in both groups, which is in accordance with another study [45]. In fact, age is the most important characteristic in IMT normality tables [26]. Furthermore, our findings suggest that there was a higher impact of age in the IMT of patients with type 1 diabetes than in the IMT of the control subjects. This higher impact may be partly due to the duration of the disease. It is important to note that in one study, it was found that CVD was the main cause of death in patients with type 1 diabetes, primarily in those who had had diabetes for more than 20 years [46].

A family history of type 2 diabetes was an important factor that influenced the IMT in both groups. The DCCT/EDIC study found that patients with type 1 diabetes and family histoy of type 2 diabetes in the intensive treatment group had a greater increase in the carotid IMT in both EDIC year 1 and 6 [47]. It is important to emphasize that in the general population, a family history of type 2 diabetes was a major determinant of endothelial dysfunction and this was able to predict the progression of carotid IMT [48,49]. Unfortunately, such an association was not evaluated in our study. However, one author failed to find any association between type 1 diabetes and endothelial dysfunction [50]. In patients with Type 1 diabetes, a family history of type 2 diabetes is associated with a metabolic profile of insulin resistance, increased carotid IMT and increased CVD risk [51,52]. One study showed that in non-obese patients with type 1 diabetes the insulin resistance was associated with a higher IMT [53]. In our study the association of IMT with family history of type 2 diabetes may reflect the insulin resistance. Another study showed an increased prevalence of a family history of type 2 diabetes in patients with type 1 diabetes and a history of coronary artery disease compared with patients with type 1 diabetes without a history of coronary artery disease [54]. Therefore, patients with type 1 diabetes and a family history of type 2 diabetes should be under an even more aggressive treatment in regards to not only glucose control but also other CVD risk factors such as blood pressure and lipid profile.

Another important CVD risk factor is the presence of renal dysfunction. The presence of renal dysfunction in type 1 diabetes patients is associated with a 10- fold increase in CVD risk compared with those patients with type 1 diabetes without renal dysfunction [55]. In our study, the association of IMT with creatinine clearance in patients with type 1 diabetes may reflect the association of CVD risk and some renal dysfunction.

The DCCT/EDIC study demonstrated that in patients with type 1 diabetes the excess weight gain was associated with higher carotid IMT, independently of treatment group [47]. In our study, the association of BMI with IMT in both patients with type 1 diabetes and in control group may reflect role of role of visceral adipose tissue on endothelial dysfunction and CVD risk as recently demonstrated [56,57].

The positive association of IMT and total cholesterol in patients with type 1 diabetes was an unexpected finding.

Carotid IMT is an established surrogate end point for CVD risk in the general population and has a strong correlation with CVD risk factors [58]. However, there are no longitudinal studies in type 1 diabetes showing a causal relationship between a higher carotid IMT and cardiovascular events, as demonstrated in the DCCT/EDIC study [28]. In this study, after 12 years of follow up, only 75 patients experienced cardiovascular events, and the authors could not demonstrate a causal effect between the increase in carotid IMT and CVD events. Despite the high CVD mortality in patients with type 1 diabetes [40], there is no specific method for CVD screening and no method to stratify CVD risk in these patients. Additionally, there is difficulty in identifying factors associated with impaired cardiac function and cardiac remodeling as suggested by the DCCT/EDIC study [59]. In this study, there was an association between the mean glycated hemoglobin over the entire follow-up period and parameters of cardiac function and structures evaluated by cardiac magnetic resonance. However, this study failed to find any difference between previously intensive versus conventionally treated patients. In a review of CVD in type 1 diabetes the authors state that in the past 40 years, there was a 70% reduction in CVD mortality in type 1 diabetes presumably because of the progress in CVD risk management. However, CVD mortality may increase approximately 50% for each 1% increase in HbA1c between the ages of 45 and 64 years [44]. The difficulty in identifying those patients with high CVD risk was illustrated by a recent case report of a 48- years-old woman with a long duration of type 1 diabetes but without evidence of microvascular complication, traditional CVD risk factors and a family history of diabetes. She had a rapidly progressive severe coronary atherosclerosis and experienced a myocardial infarction by the age of 42 [60].

The primary strength of our study resides in the fact that all IMT data were measured using a semi-automated edge-detection software and confirmed by a second independent echocardiographist. Both were blinded to the subject condition, and there was a high agreement between them. This high agreement convince us that this is a reliable and reproducible procedure that can improve the detection of patients with type 1 diabetes and increased CVD risk. Some limitations must be addressed. We studied the patients with type 1 diabetes in a single center with only internal validation. Furthermore, considering that our study was a cross-sectional one, the inference of cause-effect was not possible.

## Conclusion

Despite their young age, patients with type 1 diabetes have increased IMT, which is a marker of subclinical atherosclerosis. The CVD risk may be similar between men and women with type 1 diabetes, suggesting a loss of gender protection for CVD risk in this population. The CVD risk may even more increased in those patients with a family history of type 2 diabetes. Future prospective studies are needed to confirm the predictive value of these findings and the causal effect between the carotid IMT and CVD in patients with type 1 diabetes.

## Abbreviations

ADA: American Diabetes Association; BMI: Body mass index; CRP: C-reactive protein; CVD: Cardiovascular disease; DBP: Diastolic blood pressure; FG: Fasting glucose; HbA1c: Glycated hemoglobin; HDL: High density cholesterol; IQR: Interquartile range; IMT: Intima-media thickness; LDL: Low density cholesterol; SBP: Systolic blood pressure; sCD146: Soluble form of the membrane glycoprotein CD146; UAER: Urinary albumin excretion rate.

## Competing interests

The authors declare that they have no competing interests.

## Authors’ contributions

CRMA-J: participated in the elaboration of the objective, design of the study, acquisition of data, analysis and interpretation of data and writing the paper; ELCS: participated in recruitment of subjects, acquisition of data and collecting blood and urine samples; MdFBdM: participated in the processing and analysis of blood and urine samples. MBC: participated in the definition of how to perform the IMT measurement and in the measurement of IMT; MLGR: participated in the analysis and interpretation of data. MdBG: participated in the elaboration of the objective and design of the study, interpretation of data and writing the paper. All authors read and approved the final manuscript.

## Authors’ information

This study derived from Andrade-Junior, CRM PhD thesis. This study was funded by FAPERJ (Fundação de amparo à pesquisa do Estado do Rio de Janeiro) and CNPq (Conselho Nacional de Desenvolvimento Cientifico e Tecnológico).
